# The role of computed tomography in enterolith causing small bowel obstruction: A case series

**DOI:** 10.1097/MD.0000000000035041

**Published:** 2023-09-08

**Authors:** Jing Zhang, Ping Xie, Kefu Liu

**Affiliations:** a Department of Radiology, The Affiliated Suzhou Hospital of Nanjing Medical University, Suzhou, China.

**Keywords:** CT features, enterolith, gallstone, intestinal obstruction

## Abstract

Intestinal obstruction caused by enteroliths is an uncommon medical condition. Timely detection of the presence of enteroliths and identification of their origin can guide clinical treatment. This study aimed to present the Computed Tomography (CT) features of enterolithic ileus confirmed by surgery in 7 patients. Seven patients with surgically confirmed enterolithic ileus who were admitted to our hospital between December 2013 and December 2022 were continuously enrolled, and an abdominopelvic CT examination was performed before surgery. The imaging characteristics were then analyzed. In the transition zone of all patients with intestinal obstruction, the sharply defined intraluminal masses were found. Three of them had gallstones and 4 had primary enteroliths. All 5 enteroliths in the 4 patients with primary enteroliths were in the proximal small intestine and were low-density with gas. Additionally, 3 gallstones were present in the distal small bowel, and calcifications were observed. Simultaneously, cholecystitis and secondary cholecystoduodenal fistula were observed in all 3 patients with gallstones. Compared to gallstones, primary enteroliths tend to be higher positioned, less dense, and accompanied by gas. CT examination is very important, as it allows accurate identification, location, diagnosis, and identification of complications of the different types of enteroliths to provide a basis for surgery.

## 1. Introduction

Small bowel obstruction (SBO) remains the leading cause of emergency room visits, inpatient admissions, and emergency surgery. Adhesions are by far the most common cause, explaining 60% to 70% of all SBO.^[1]^ Intestinal obstruction caused by the presence of enteroliths is an uncommon medical condition.^[2,3]^ It is important to formulate a treatment plan for patients if SBO caused by intestinal stones can be quickly identified. However, the clinical and laboratory signs of SBO lack sensitivity for identifying the causes. Imaging studies have provided valuable information in this regard.

Plain abdominal roentgenograms are the first step in identifying enteroliths and can detect enteroliths containing a higher proportion of calcium salts that are more radiopaque.^[4]^ However, the sensitivity of plain abdominal roentgenograms for detecting enteroliths is low, and their ability to locate obstructions is imprecise.^[5]^

Computed tomography (CT) may provide 2- or 3-dimensional imaging and increase the detection yield of radiolucent stones. CT scans may also help in identifying the number of enteroliths, their exact location, and narrowing the focus to the culprit stone.^[4]^ In addition, radiological imaging may assist in establishing the underlying pathology of the intestinal tract that leads to a stone formation, or is responsible for stone trapping and clinical obstruction.

According to scientific literature, enteroliths can be divided into primary and secondary enteroliths.^[2]^ Primary enteroliths can be further subdivided into “true” and “false” subtypes. Due to the rarity of enterolithic ileus, radiologists, especially young radiologists, lack awareness of the CT features of enteroliths, let alone the differences between the subtypes. In addition, studies of enteroliths causing SBO have mostly been isolated case series or reports in the recent literature.^[4–8]^

Thus, we attempted to identify the CT signs of enterolithic ileus confirmed by surgery in 7 patients and the differences between the 2 types of enteroliths. The results of this study will hopefully provide a basis for CT signs to accurately predict SBO caused by enteroliths.

## 2. Materials and Methods

### 2.1. Study population

Seven patients with enterolithic ileus who underwent surgery at the affiliated Suzhou Hospital of Nanjing Medical University between December 2013 and December 2022 were included in this study. Patients with enterolithic ileus, based on surgical records and results, were included in the study, and those with other types of ileus were excluded.

### 2.2. CT scans

Abdominopelvic CT examinations were performed using 3 different CT machines: a 16 slice multi detector CT (Brilliance, Philips), a 64 slice multi detector CT (Ingenuity, Philips), and a 256 slice multi detector CT (Brilliance iCT; Philips). The scanning parameters are listed in Table 1. The patients were examined in the supine position. The scanning range was from the diaphragm to the lower border of the pubic symphysis. After scanning, the original image was reconstructed, the thickness of the reconstruction layer was 1mm, and the reconstructed image was uploaded to a post-processing workstation for multiplane reconstruction.

**Table 1:** CT technical parameters.

	Philips 16 slice	Philips 64 slice	Philips brilliance iCT 256 Slice
kV	120	120	120
mA	283,425	30	30
Field of view (mm)	500	500	500
Slice thickness (mm)	5	5	5
Reconstructed thickness (mm)	1	1	1

CT = computed tomography.

### 2.3. Image interpretation

CT scans were reviewed by 2 experienced radiologists (7 and 10 years of experience with abdominal CT) who were blinded to both the clinical history and surgical data and reached a consensus on all imaging analyses. The following data were recorded: Shape of the enterolith: round, oval, irregular; Density: high-density, low-density, unevenly high-density, and unevenly low-density compared with bowel wall^[9]^; Location: jejunum, ileum, junction of jejunum and ileum; Number and size of enteroliths; Conditions of small bowel obstruction: degree (mild: 3–4cm, moderate to severe: >4 cm), gas-liquid level (present/absent), thickened mesentery (present/absent); Intestinal fistula (present/absent); Peritoneal effusion (present/absent); Gallbladder abnormalities: gallstones in the gallbladder, gas in the gallbladder, cholecystitis, and gas in the bile duct (present/absent).

### 2.4. Stastical analysis

The age of the patients and the sizes of the gallstones and primary enteroliths were averaged using SPSS v.26 (IBM, Chicago, IL). The frequencies of most CT features were determined using direct counting.

## 3. Results

The mean age of all 7 patients (5 males, 2 females) was 75.29 ± 7.70 years old, ranging from 67 to 91 years. The clinical presentation includes abdominal pains, distention, nausea, vomiting and constipation.

**Figure 1.**
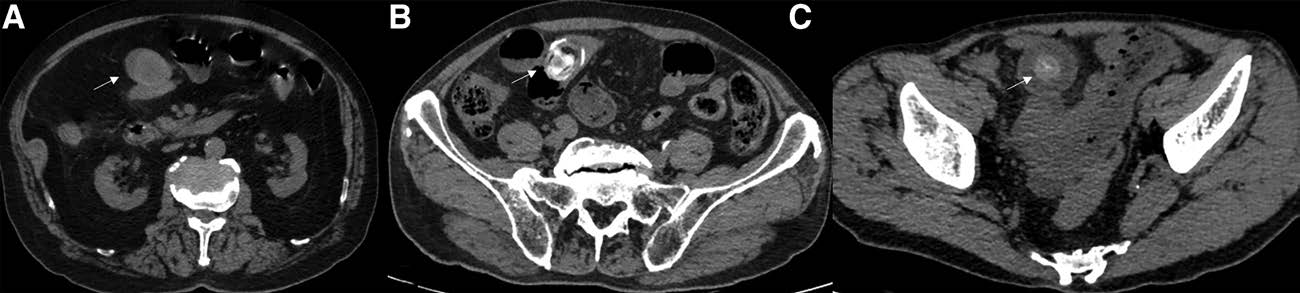
CT scans in the axial plane show three gallstones in the small bowel segment (arrow). (A) A round, high-density stone of a 91-year-old-man in ileum. (B) An oval stone of a 74-year-old-man at the junction of jejunum and ileum appears to be high-density mixed with low-density. (C) A round, high-density stone of a 71-year-old-man in ileum. CT = computed tomography.

**Figure 2.**
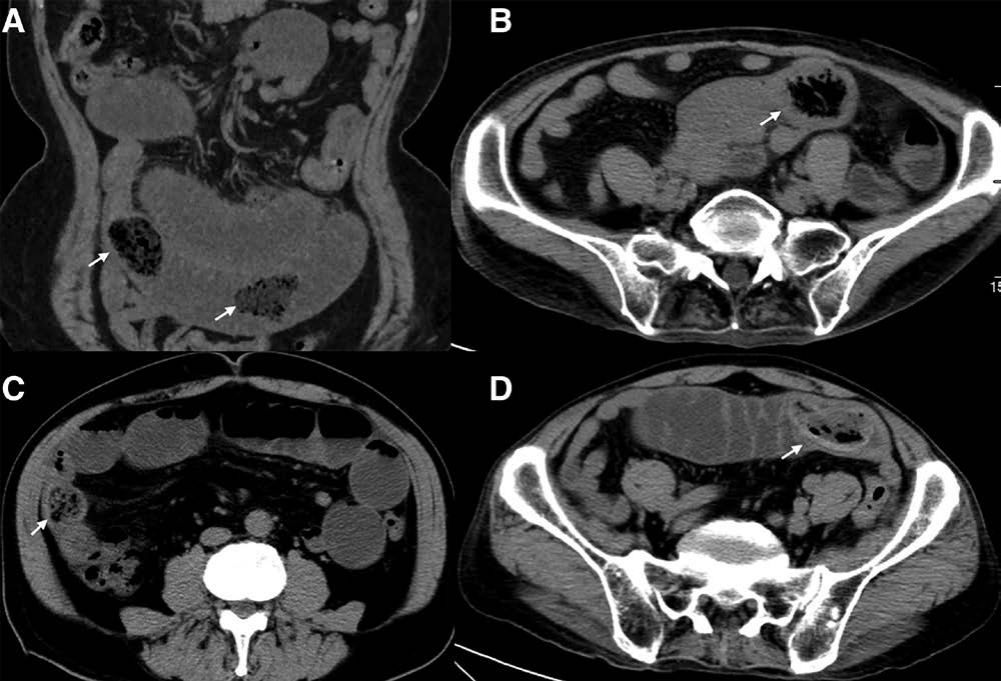
CT scans in the coronal and axial planes show five primary enteroliths that appear to be unevenly low-density with a mottled gas appearance (arrow). (A) Two stones of a 67-year-old-femal in the jejunum, one appears oval and the other irregular. (B) A round-like stone of a 72-year-old-femal located in the jejunum. (C) A round-like stone of a 74-year-old-man at the junction of jejunum and ileum. (D) An oval stone of a 78-year-old-man located in the jejunum with a slightly higher density ring. CT = computed tomography.

In all patients with small bowel obstruction, dilated small bowel loops proximal to the transition zone were observed. Sharply defined intraluminal masses were found in the transition zone, of which 3 cases were surgically confirmed to be gallstones (Fig. 1), and 4 cases were confirmed to be primary enteroliths (Fig. 2). In 1 patient with a primary enterolith, an additional small stone with a similar appearance was detected within a dilated loop proximal to the transition zone.

In patients with primary enteroliths, only 1 enterolith was located at the junction of the jejunum and ileum, whereas the rest were located in the jejunum. Among the 5 enteroliths in the 4 patients with primary enteroliths, 2 were oval, 2 were round, and only 1 was irregular in shape. All enteroliths were unevenly low-density with a mottled gas appearance and were shaped like a honeycomb. Only 1 enterolith had a slightly higher peripheral density ring. In another case, punctate calcification was observed in the enterolith. Intestinal fistulas were absent in all the patients with primary enteroliths.

**Figure 3.**
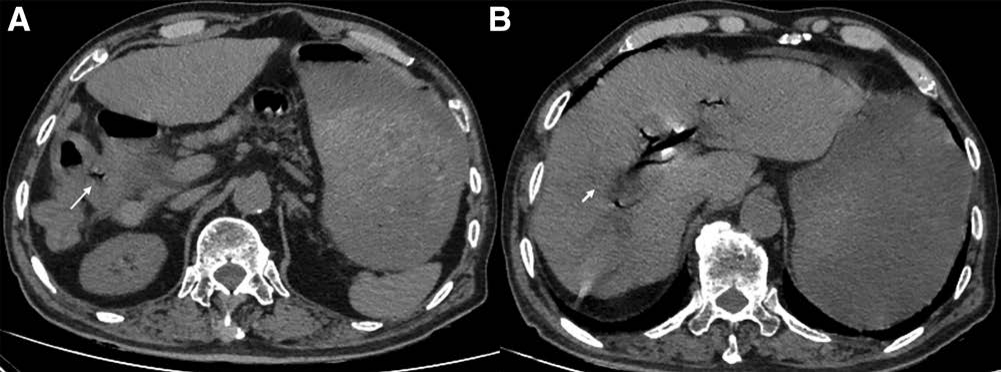
Biliary-enteric fistula in a 74-year-old-man. (A) CT scan in the axial plane demonstrates a communication between gallbladder and duodenum (arrow) suggesting biliary-enteric fistula. (B) CT scan in the axial plane shows gas in the gallbladder (short arrow). CT = computed tomography.

All gallstones were in the ileum except in 1 case at the junction of the jejunum and ileum. CT scans of 3 patients with gallstones revealed cholecystitis and secondary biliary-enteric fistula (Fig. 3) with gas in the gallbladder. One patient had gallstones in the gallbladder and the other had gas in the bile duct. Two gallstones were round, and only 1 was oval. All of these were high-density calcifications, with only 1 mixed low-density.

In both groups, 1 case of moderate to-severe bowel dilation was observed. One patient with a primary enterolith had peritoneal effusion, which in 2 patients with gallstones. Mesenteric thickening was observed in all the patients. The CT characteristics of all patients are shown in Table 2.

**Table 2:** The CT characteristics of gallstones and primary enteroliths.

Parameter	Primary enterolith	Gallstone
Number	5	3
Shape		
Round	2	2
Oval	2	1
Irregular	1	0
Density		
High-density	0	2
Low-density	0	0
Unevenly high-density	0	1
Unevenly low-density	5	0
Location		
Jejunum	4	0
Ileum	0	2
The junction of jejunum and ileum	1	1
Size (cm)	4.5	4.2
Degree of small bowel obstruction		
Mild	3	2
Moderate to severe	1	1
Gas-liquid level	3	3
Thickened mesentery	4	3
Complication		
Biliary-enteric fistula	0	3
Peritoneal effusion	1	2
Gallstones in the gallbladder	0	1
Gas in the gallbladder	0	3
Cholecystitis	0	3
Gas in the bile duct	0	1

CT = computed tomography.

## 4. Discussion

CT plays a crucial role in the diagnosis of SBO caused by enteroliths. Clinical assessment is often difficult because of atypical clinical symptoms.^[10]^ In contrast, CT imaging can help determine whether an enterolith is present, locate the site of the enterolith, identify the cause, and identify complications such as perforation.^[1]^

Intra-abdominal adhesions and intestinal hernias are the most common causes of SBO. Enteroliths causing SBO are rare and often poses a challenge to gastroenterologists for diagnosis. The diagnosis of SBO requires the identification of the transition zone, which is the short segment area between the dilated proximal bowel and decompressed distal bowel. The discovery of sharply defined intraluminal masses in the transitional zone requires consideration of enterolithic ileus. Conversely, the transition zone like a “beaked” in adhesive small bowel obstruction.^[11]^ CT may show a fat notch sign in adhesive SBO, which is caused by the extraluminal compression of the band in the transition zone. In addition, angulation, kinking, and/or twisting of the bowel can be observed in the adhesions. Furthermore, the presence of inflammatory or tumoral thickening of the bowel wall or the identification of an extrinsic mass ruled out enterolithic ileus.

According to scientific literature, enteroliths can be divided into primary and secondary enteroliths.^[2]^ CT may assist in establishing the underlying pathology of the intestinal tract that leads to stone formation, or is responsible for stone trapping and clinical obstruction. Primary enteroliths always occur in older patients, especially those with anatomical alterations of the gastrointestinal tract, such as harmless anatomical variations (diverticula, duplication cysts) or pathological conditions (strictures).^[12]^ The most common type of secondary enteroliths is gallstones, which enter the intestinal tract through a biliary-enteric fistula. In our study, of the 4 patients with primary enteroliths, 1 was a 67-year-old female with a history of gastrectomy. A known duodenal diverticulum presented in a 74-year-old male patient. A cholecystoduodenal fistula was found in all 3 patients with gallstone ileus.

Primary enteroliths, which are formed within the gastrointestinal tract, can be further subdivided into the “true” and “false” subtypes. The true primary enteroliths reported in recent literatures were all located in the distal small intestine, with high-density calcification.^[4–6,12–17]^ This is because they may contain calcium phosphate, calcium oxalate, and calcium carbonate.^[17,18]^ Mottled gas appearance is a characteristic CT finding in false primary enteroliths. Some cases can be accompanied by an encapsulating wall or calcification.^[9,19,20]^ All of these features are consistent with those of our primary small intestines. Unfortunately, the detailed contents of the stones were not be identified in our study. Although CT cannot determine the exact classification of stones, it has a certain guiding significance in the treatment of patients.

Gallstones are primarily composed of bilirubin, bile salts, and cholesterol.^[21]^ All 3 radiopaque gallstones in our study showed calcification. However, some gallstones composed of cholesterol and bile salts are radiolucent,^[22]^ in which case CT was not helpful for detecting stones. One case reported by Tiong et al^[23]^ displayed symptoms of bowel obstruction; however, blood tests and imaging were not definitive. Although a secondary cholecystoduodenal fistula was suggested on CT, no stones were found within the small bowel and the transition points were not clearly defined. If a high index of suspicion for gallstone ileus is given, magnetic resonance imaging may be the gold standard imaging modality.

In this study, we found that primary enteroliths were more frequently located in the jejunum than were gallstones. As the literature shows,^[2]^ choleic acid enteroliths are typically found in the proximal small intestine due to the need for a lower pH, which may be 1 reason for the location of our primary enteroliths. Besides, since all of the patients in our study were elderly and had slower peristalsis, the enteroliths were more likely to obstruct the proximal small intestine. In addition, primary enteroliths are all unevenly low-density, whereas gallstones are all high-density.

Our study had some limitations. First, we only collected 7 cases of enterolith ileus due to its rarity. A larger study population is needed to further support our findings. Second, the CT characteristics of the primary enteroliths may be influenced by their position in the small intestine. The contents of distal primary enteroliths could not be determined because the primary enteroliths in this investigation were all in the proximal small intestine. Additionally, the participants in this study were exclusively senior patients. Further research is required to understand the CT characteristics of the enteroliths in young and middle-aged patients. Further studies are necessary to address these limitations.

## 5. Conclusion

In conclusion, by combining the different CT features of primary enteroliths and gallstones, our study demonstrates that CT plays a pivotal role in evaluating patients with enterolithic ileus. CT can not only accurately identify most enteroliths but also locate the site of the enterolith, identify the cause, and look for complications. This information will be useful for determining the best therapeutic strategy at the earliest opportunity.

## Author contributions

**Conceptualization:** Jing Zhang, Kefu Liu.

**Data curation:** Jing Zhang, Ping Xie, Kefu Liu.

**Formal analysis:** Jing Zhang, Kefu Liu.

**Investigation:** Jing Zhang.

**Methodology:** Kefu Liu.

**Supervision:** Kefu Liu.

**Writing – original draft:** Jing Zhang.

**Writing – review & editing:** Ping Xie, Kefu Liu.

## References

[1] Zins M Millet I Taourel P. Adhesive small bowel obstruction: predictive radiology to improve patient management. Radiology. 2020;296:480–92. 32692296 10.1148/radiol.2020192234

[2] Gurvits GE Lan G. Enterolithiasis. World J Gastroenterol. 2014;20:17819–29. 25548480 10.3748/wjg.v20.i47.17819 PMC4273132

[3] Santos SD Louro J Costa Almeida CM. Gallstone ileus: a rare cause of mechanical bowel obstruction. Cureus. 2023;15:e35588. 37007418 10.7759/cureus.35588 PMC10062434

[4] Jadib A Tabakh H Chahidi El Ouazzani L. Primary true enterolithiasis: a rare cause of acute small bowel obstruction. Radiol Case Rep. 2022;17:610–4. 34987690 10.1016/j.radcr.2021.11.032 PMC8703179

[5] Molla YD Tassew MT Abebe TA. Enterolithiasis: an unusual cause of large bowel obstruction, a case report. Int J Surg Case Rep. 2023;103:107889. 36638585 10.1016/j.ijscr.2023.107889 PMC9843248

[6] Nastos C Giannoulopoulos D Georgopoulos I. Large enterolith complicating a meckel diverticulum causing obstructive ileus in an adolescent male patient. Case Rep Surg. 2017;2017:1871434. 29391964 10.1155/2017/1871434 PMC5748096

[7] Takayama N Takagaki Y. Afferent loop obstruction induced by undigested food (phytobezoar) treated through endoscopic fragmentation with biopsy forceps: a case report. Int J Surg Case Rep. 2023;107:108365. 37267790 10.1016/j.ijscr.2023.108365 PMC10310906

[8] Goh SL Steen C Wong E. Small bowel obstruction secondary to a plastic bezoar. BMJ Case Rep. 2022;15:e251438. 10.1136/bcr-2022-251438 PMC9710328 36446472

[9] Gao X Hua Y Meng H. The value of MSCT in evaluating the passability of bezoar by conservative treatment for bezoars-induced small bowel obstruction. Abdom Radiol (NY). 2023;48:236–43. 36242605 10.1007/s00261-022-03700-4

[10] Schmidt SA Beer M Vogele D. Update: small bowel diseases in computed tomography and magnetic resonance imaging. Radiologie (Heidelb). 2023;63:435–40. 37016034 10.1007/s00117-023-01139-2

[11] Tai FWD Sidhu R. Small bowel obstruction: what a gastroenterologist needs to know. Curr Opin Gastroenterol. 2023;39:234–41. 36976860 10.1097/MOG.0000000000000924

[12] Perathoner A Kogler P Denecke C. Enterolithiasis-associated ileus in Crohn’s disease. World J Gastroenterol. 2012;18:6160–3. 23155347 10.3748/wjg.v18.i42.6160 PMC3496895

[13] Hirata T Kawakami H Kubota Y. Huge enterolithiasis in crohn’s disease. Clin Gastroenterol Hepatol. 2019;17:e141. 29857142 10.1016/j.cgh.2018.05.033

[14] de Leon Castorena E de Leon Castorena MD. Intestinal stones: a rare cause of bowel obstruction. SAGE Open Med Case Rep. 2019;7:2050313X1984983–19849837. 10.1177/2050313X19849837 PMC6537081 31205711

[15] Couto JP Rodrigues AC. An unusual cause of abdominal pain. Gastroenterology. 2021;161:e9–e10. 10.1053/j.gastro.2021.02.031 33610529

[16] Ferrari-Light D Shuchleib A Ricci-Gorbea J. Bile salt enterolith: an unusual etiology mimicking gallstone ileus. Case Rep Surg. 2018;2018:8965930. 30662783 10.1155/2018/8965930 PMC6313996

[17] Sharma O Mallik D Ranjan S. Enterolith causing small bowel obstruction: report of a case and review of literature. Clin Exp Gastroenterol. 2022;15:101–4. 35859713 10.2147/CEG.S369640 PMC9292452

[18] Tewari A Weiden J Johnson JO. Small-bowel obstruction associated with Crohn’s enterolith. Emerg Radiol. 2013;20:341–4. 23392960 10.1007/s10140-013-1107-y

[19] Zissin R Osadchy A Gutman V. CT findings in patients with small bowel obstruction due to phytobezoar. Emerg Radiol. 2004;10:197–200. 15290490 10.1007/s10140-003-0297-0

[20] Chen YC Liu CH Hsu HH. Imaging differentiation of phytobezoar and small-bowel faeces: CT characteristics with quantitative analysis in patients with small- bowel obstruction. Eur Radiol. 2015;25:922–31. 25417124 10.1007/s00330-014-3486-1

[21] Beji H Chtourou MF Zribi S. Gallstone ileus: a case report and review of the literature. Int J Surg Case Rep. 2023;106:108221. 37075501 10.1016/j.ijscr.2023.108221 PMC10201841

[22] Pinheiro JL Logrado A Aveiro D. Synchronous gallstone ileus and bouveret’s syndrome: a report of two rare concurrent complications of gallstone disease. Cureus. 2023;15:e35672. 37012966 10.7759/cureus.35672 PMC10066062

[23] Tiong J Grant K Shilton H. The role of magnetic resonance imaging in a difficult case of gallstone ileus. Cureus. 2022;14:e33038. 36721596 10.7759/cureus.33038 PMC9881392

